# Efficacy and safety of labetalol vs. Hydralazine in pregnant women with severe hypertension

**DOI:** 10.12669/pjms.42.1.12247

**Published:** 2026-01

**Authors:** Natasha Bushra, Naila Fayyaz, Tayyiba Wasim

**Affiliations:** 1 Natasha Bushra, (MRCOG) Department of Obstetrics and Gynecology, Post Graduate Medical Institute, Ameer-ud-Din Medical College, Lahore, Pakistan; 2 Naila Fayyaz, (MBBS) Department of Obstetrics and Gynecology, Services Institute of Medical Sciences Lahore, Lahore, Pakistan; 3 Tayyiba Wasim, (FRCOG) Department of Obstetrics and Gynecology, Principal and Head of Department of Obstetrics and Gynecology, Allama Iqbal Medical College, Lahore, Pakistan

**Keywords:** Hydralazine, Labetalol, Pregnancy, Pre-eclampsia, Severe Hypertension

## Abstract

**Objective:**

The study aims to evaluate the effectiveness of labetalol versus hydralazine in managing severe hypertension during pregnancy.

**Methodology:**

A quasi-experimental study was conducted in the unit of the Department of Gynecology, Services Hospital Lahore, from July 30, 2021, to November 30, 2022. Recruitment of 320 pregnant women diagnosed with severe hypertension were allocated into two treatment groups. Group-A (n=160) received IV labetalol in incremental doses up to 80 mg at 20 minutes interval with maximum cumulative dose of 300 mg, and Group-B (n=160) was administered IV hydralazine in 5mg dose over five minutes, repeated at 20 minutes interval with maximum of five doses. Blood pressure and pulse were monitored every 10 minutes, with follow-up lasting for one hour. Efficacy was defined as achieving target BP ≤140/100 mmHg. Data was analyzed using SPSS version 23.

**Results:**

The target BP was achieved in 248 (77.50%) women, with 134 (83.8%) in the labetalol Group-And 114 (71.2%) in the hydralazine group (p=0.007). Hydralazine achieved BP control faster (50.25 ± 20.43 min vs. 75.34 ± 35.67 min, p=0.001), with fewer doses required (1.85 ± 0.92 vs. 3.68 ± 1.45, p=0.0001).

**Conclusion:**

Labetalol showed better overall efficacy, while hydralazine provided faster BP control with higher adverse effects. Labetalol is recommended for sustained BP management, and hydralazine for rapid BP reduction.

## INTRODUCTION

Gestational hypertension is a frequent hypertensive problem of pregnancy that influences a significant proportion of pregnancies worldwide. It poses considerable risks to both maternal and fetal health if not appropriately managed.^1^ Complications incorporate premature birth, growth restriction in offsprings, placental complications and progression to severe hypertension, which can result in life-threatening conditions such as eclampsia and stroke. Hypertension complicates up to 10% of pregnancies and contributes to more than 60,000 maternal deaths annually, with more than 90% occurring in low-income countries.^2^

United Kingdom documents that proteinuric hypertension is recognized as the second leading contributor of direct maternal mortality and perinatal complications.^3^ Similarly United States accounts for six to nine maternal deaths and over 175 infant fatalities each year.^4^ In Pakistan, hypertensive disorders, including preeclampsia and eclampsia, contribute to approximately 10% of maternal deaths, with tertiary care hospitals reporting mortality rates of 4% to 8%.^5^ These disparities highlight the urgent need for standardized screening, timely interventions, and improved antenatal care to reduce adverse outcomes.^6^,^7^

NICE guidelines categorize hypertension in pregnancy as (a) gestational hypertension, which develops after 20 weeks of gestation in previously normotensive women (b) severe hypertension is defined as systolic BP ≥160 mmHg or diastolic BP ≥110 mmHg, typically in women with pre-existing hypertension or occurring before 20 weeks of gestation (c) preeclampsia, characterized by Proteinuric hypertension which can persist postpartum; and (d) eclampsia, involving hypertensive-related seizures. Chronic hypertension, diagnosed before 20 weeks, can sometimes be complicated by superimposed preeclampsia.^8^

Preeclampsia can result in severe maternal complications such as seizures, stroke, ischemic heart disease, acute kidney injury and HELLP syndrome. It also poses significant fetal risks, including restriction of fetus, intrapartum heart changes in baby may lead to stillbirth.^9^ To deter these adversities, timely administration of immediate-effect blood pressure lowering medications is crucial to attain prompt systemic pressure control while mitigating pitfalls such as maternal hypoperfusion and non-reassuring fetal heart rate.^4^ Once blood pressure is adequately controlled, delivery decisions depend on duration of pregnancy and the presence of maternal or fetal risks. Pregnancy duration near thirty seven weeks, immediate delivery is usually recommended, whereas in cases of extreme prematurity, observation based approach potentially deemed in specialized centers to optimize offspring implications.^10^

Several hypertension treatment agents are available for managing severe hypertension in pregnancy.^4^ Despite multiple international and national studies evaluating their efficacy, many clinicians in low- and middle-income countries, including Pakistan, continue to follow Western treatment protocols. The most commonly used medications for rapid BP reduction include labetalol, hydralazine, and nifedipine, with no universal consensus on the most effective option.^9^

Intravenous labetalol has gained preference globally due to its combined alpha- and beta-blocking properties, offering a controlled and gradual BP reduction with minimal side effects.^11^ On the other hand, IV hydralazine, a direct vasodilator, is favored for its rapid onset of action, although concerns remain regarding adverse effects such as reflex tachycardia and maternal hypotension.^12^ Studies conducted in different settings, including Pakistan, have produced mixed results on the superiority of these drugs, emphasizing the need for local, evidence-based data to guide clinical decisions.^12^

In Pakistani healthcare settings, both IV labetalol and hydralazine are used to manage severe hypertension during pregnancy, with labetalol being preferred in tertiary care hospitals due to its better safety profile.^13^ However, the choice of anti-hypertensive therapy remains individualized based on patient-specific factors and clinical presentation.^11^

Although several Pakistani studies have compared labetalol, hydralazine and even nifedipine in pregnancy, most have important limitations, including, mixed cohorts of pre-eclampsia and eclampsia, and lack of stratified analysis by demographic or clinical characteristics.^14^-^16^ This study adds novelty by evaluating both efficacy and safety of IV labetalol versus IV hydralazine in a clearly defined cohort of women with severe gestational hypertension, and by incorporating subgroup analyses based on age, parity, and BMI. By providing context-specific, evidence-based data, this study aims to contribute to improved clinical decision-making and enhance treatment strategies to optimize mother and offspring implications.

## METHODOLOGY

The quasi-experimental study was carried out in the Department of Gynecology, Services Hospital Lahore, from July 30, 2021, to January 30, 2022. The study aimed to evaluate the efficacy of intravenous labetalol versus IV hydralazine in the treatment of severe gestational hypertension. Total of 320 pregnant women diagnosed were enrolled in the study based on predefined criteria. Participants were allocated into two treatment groups using a non-probability consecutive sampling technique. All eligible women presenting with severe gestational hypertension during the study period were enrolled consecutively and assigned to Group A (labetalol) or Group-B (hydralazine) ensuring that every eligible patient was included without randomization. Group-A (n=160) who were treated with IV labetalol in escalating doses of 20, 40, 80, 80, and 80 mg i.e., maximum dose of 300 mg administered at 20 minutes interval until the target blood pressure of ≤140/100 mmHg was documented. Group-B (n=160) was given intravenous hydralazine in a dose of 5 mg over 5 minutes. The dose of hydralazine was given after every 20 minutes up to cumulative dose 15 mg till required blood pressure is achieved.

Pregnant women with duration of pregnancy ≥28 weeks with diagnosed severe gestational hypertension (systolic BP ≥160 mmHg and/or diastolic BP ≥110 mmHg), singleton pregnancies and no known history of chronic hypertension prior to pregnancy were enrolled. Women with a known history of asthma, chronic obstructive airway disease, cardiac conduction abnormalities, bradyarrhythmias, or any contraindication to labetalol or hydralazine were not included in the study. This ensured appropriate patient selection and minimized the risk of medication-related complications. Blood pressure and pulse rate were monitored at 10-minute intervals throughout the study. If systolic BP remained ≥160 mmHg, additional doses were administered until the required BP was documented. Patients were followed up for 1-hour post-treatment to assess efficacy and potential adverse effects, such as vomiting and hypotension. Primary Outcome was achievement of target BP (≤140/100 mmHg) within one hour of intervention and secondary Outcomes were time period required to normalize blood pressure, doses required and incidence of adverse effects (e.g., headache, nausea, hypotension) on mother. If the target blood pressure was not achieved despite the maximum allowable dose of the assigned medication, the patient was considered a non-responder. As per unit protocol, such patients were escalated to second-line management, which included switching to the alternative antihypertensive agent and planning delivery if indicated. These cases were managed according to institutional guidelines and were not included further in comparative efficacy analysis.

### Ethical approval:

It was taken from ethical review board of Services Institute of Medical Science, IRB/2019/591/SIMS, dated October 21, 2019.

### Statical analysis:

All data were recorded in a structured proforma and subsequently entered into SPSS version 23 for analysis. Descriptive statistics such as mean ± standard deviation (SD) was used to summarize continuous variables, while categorical variables were expressed as frequencies and percentages. The chi-square test was used to compare categorical variables, and an independent t-test was applied for continuous variables to determine statistically significant differences between the two groups. A p-value of <0.05 was considered statistically significant.

## RESULTS

A total of 320 mothers diagnosed with severe gestational hypertension were enrolled in the study and allocated into two groups. The mean age of participants in the labetalol group was 30.5 ± 4.2 years, while in the hydralazine group, it was 29.8 ± 4.5 years. Group-A (n=160) received IV labetalol, while Group-B (n=160) received IV hydralazine. The baseline features of participants were comparable between both groups.

The Table-I presents a comparison of the efficacy of both groups in managing blood pressure stratified by age, parity, and body mass index (BMI). A significant difference was observed in participants aged >30 years, with labetalol achieving blood pressure control in 88.5%, compared to 61.5% with hydralazine (p=0.002). Among multiparous women, labetalol demonstrated significantly higher efficacy (77.8%) compared to hydralazine (63.1%) (p=0.039), indicating its superior performance in this subgroup. In overweight and obese participants, labetalol showed significantly better efficacy (83.7%) compared to hydralazine (70.8%) (p=0.011). These results suggest that labetalol is more efficient in controlling severe hypertension in older, multiparous, and overweight/obese women compared to hydralazine.

**Table-I:** Comparison of efficacy between study groups stratified by age, parity & BMI.

Efficacy		Study Groups		Total	p-value
		Labetalol	Hydralazine		
Age (years)	≤30	Yes	88(81.5%)	82(75.9%)	170(78.7%)	0.319
		No	20(18.5%)	26(24.1%)	46(21.3%)	
	>30	Yes	46(88.5%)	32(61.5%)	78(75.0%)	0.002
		No	6(11.5%)	20(38.5%)	26(25.0%)	
Parity	Null & primary	Yes	71(89.9%)	61(80.3%)	132(85.2%)	0.092
		No	8(10.1%)	15(19.7%)	23 (14.8%)	
	Multiple	Yes	63(77.8%)	53(63.1%)	116(70.3%)	0.039
		No	18(22.2%)	31(36.9%)	49(29.7%)	
BMI	Normal	Yes	11(84.6%)	17(73.9%)	28(77.8%)	0.428
		No	2(15.4%)	6(26.1%)	8(22.2%)	
	Overweight & Obese	Yes	123(83.7%)	97(70.8%)	220(77.5%)	0.011
		No	24(16.3%)	40(29.2%)	64(22.5%)	

The overall efficacy, defined as achieving the intended blood pressure within the follow-up period, was achieved in 248 (77.50%) participants. In the labetalol group, 134 out of 160 women (83.8%) achieved the target BP, compared to 114 out of 160 women (71.2%) in hydralazine group, showing a statistically proven difference (p=0.007).

The comparison between both groups in terms of time to reach the required value of blood pressure, cumulative doses required, and failure rates is summarized in Table-II. The average time required to normalize BP was significantly lower in the hydralazine group (50.25 ± 20.43 minutes) compared to the labetalol group (75.34 ± 35.67 minutes, p=0.001). Similarly, the mean cumulative doses required to achieve required BP was significantly lower in the Group-B (1.85 ± 0.92 doses) compared to the labetalol group (3.68 ± 1.45 doses, p=0.0001).

**Table-II:** Comparison of Time to Normalize BP, Number of Doses, and Failure Rate.

	Hydralazine Group (n=160)	Labetalol Group (n=160)	p-value
Time to achieve target BP (minutes)	50.25 ± 20.43	75.34 ± 35.67	0.001
Total doses required (mean ± SD)	1.85 ± 0.92	3.68 ± 1.45	0.0001
Single dose success (n, %)	78 (48.5%)	47 (29.7%)	0.02
2-3 doses success (n, %)	60 (37.5%)	83 (51.9%)	0.03
4-5 doses success (n, %)	22 (13.8%)	30 (18.4%)	0.15
Failure rate (n, %)	46 (28.8%)	26 (16.2%)	0.007

**Fig.1:**
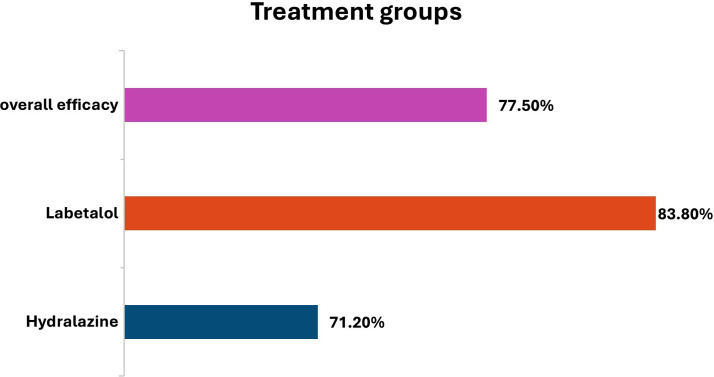
Efficacy among pregnant patients.

**Fig.2:**
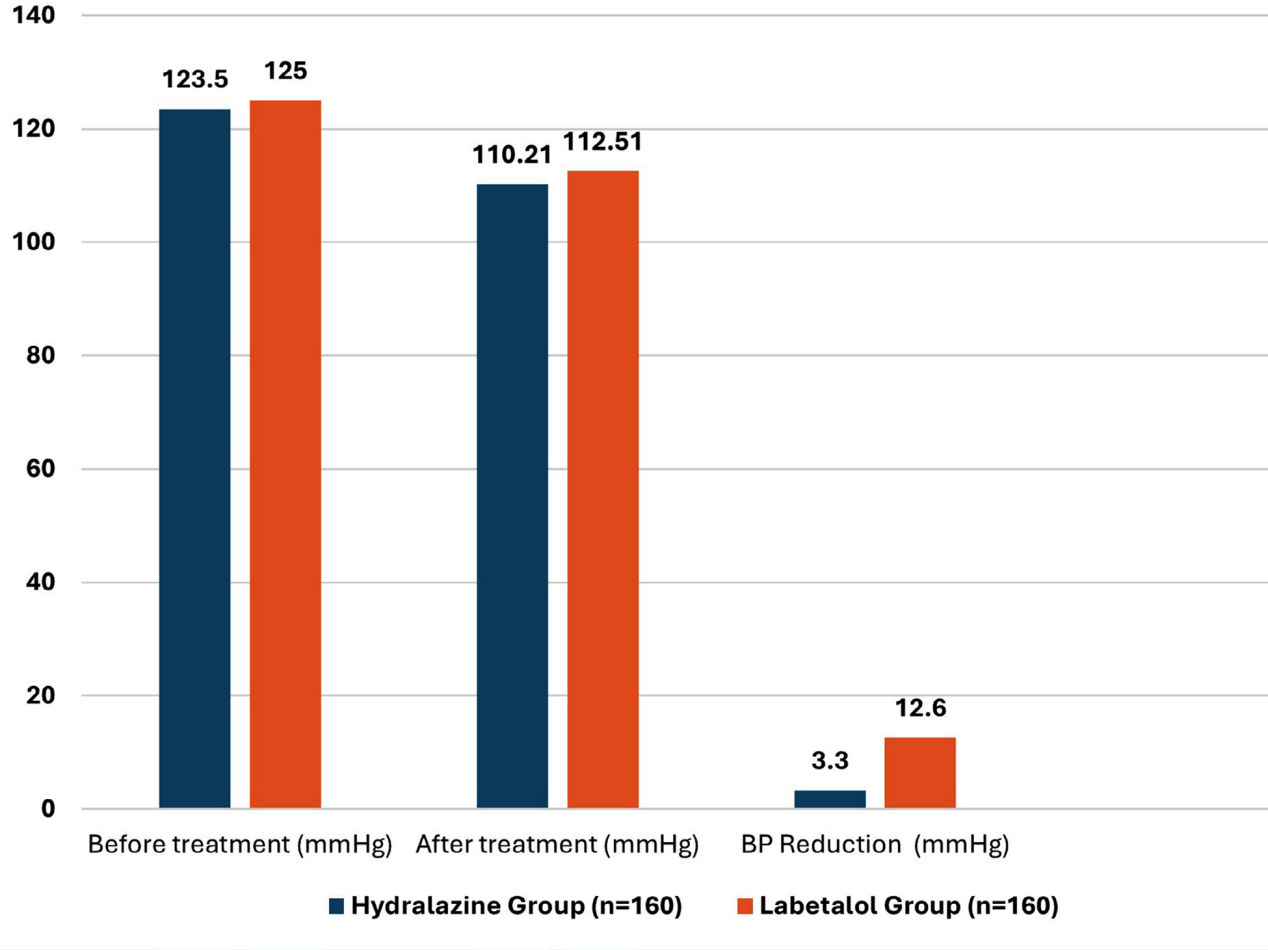
Mean arterial blood pressure changes before and after one-hour treatment.

Fig.1 provides efficacy of drugs among pregnant patients and Fig.2 provides an overview of the blood pressure response to both medications, highlighting the comparative effectiveness of both groups in declining MAP. MAP reduction was slightly greater in the hydralazine group, but the difference was not proven significant (p=0.456). For single-dose effectiveness, 48.5% of women in the Group-B reached to intended BP with a single dose compared to 29.7% in the Group-A (p=0.02). However, labetalol was more effective in cases requiring 2-3 doses, with 51.9% success compared to 37.5% in the hydralazine group (p=0.03). There was no significant difference among Group-A and B in achieving target BP within 4-5 doses (p=0.15).

The failure rate, which is inability to maintain required BP within the treatment protocol, was prominently higher in the hydralazine group (28.8%) compared to the labetalol group (16.2%, p=0.007), indicating better overall efficacy of labetalol in achieving sustained BP control. Females treated with labetalol delivered via vaginal route whereas cesarean section rates were higher in the hydralazine group, but the differences were not significant (chi-square 0.467, p-value is 0.792) Table-III. Comparison of adverse side effects between both Groups are tabulated in Table-IV showing headache was a prevalent side effect in Group-B where mothers were treated with hydralazine (p=0.045).

**Table-III:** Comparison of mode of delivery between hydralazine and labetalol groups.

Mode of delivery	Hydralazine Group (n=160)	Labetalol Group (n=160)	p-value
Vaginal delivery	90(56.3%)	95(59.4%)	0.543
Cesarean section	60(37.5%)	55 (34.4%)	0.487
Instrumental delivery	10(6.2%)	10(6.2%)	1.000
Chi-Square value	0.467		
P value	0.792		

**Table-IV:** Comparison of Adverse side effects Between Hydralazine and Labetalol Groups.

Adverse side effects	Hydralazine Group (n=160)	Labetalol Group (n=160)	p-value
Headache	45(28.1%)	30(18.8%)	0.543
Nausea	35(21.9%)	25 (15.6%)	0.487
Hypotension	20(12.5%)	12(7.5)	
Tachycardia	15(9.4%)	10(6.3%)	1.000
Injection site area	9(5.9%)	5(3.1%)	0.156

## DISCUSSION

Severe gestational hypertension remains a critical challenge in obstetric care, requiring rapid and sustained blood pressure (BP) control to prevent poor outcome in mothers and offsprings.^17^ This study aimed to compare the efficacy of intravenous (IV) labetalol and IV hydralazine in to see BP (≤140/100 mmHg) within one hour of intervention. Our findings indicate that while both medications are effective, labetalol demonstrates superior overall efficacy, whereas hydralazine offers faster BP reduction, which is consistent with study in the International Journal of Clinical Medicine and Case Reports.^18^ Similarly, meta-analysis found these blood pressure lowering drugs are optimal drugs for lowering mean arterial pressure and at the same time labetalol had minimal risks in mothers.^19^ These studies support our observation of labetalol’s superior overall efficacy and hydralazine’s rapid BP-lowering effect.

In our study, target blood pressure (BP) was documented in 83.8% of women in the labetalol group compared to 71.2% in the hydralazine group (p=0.007), indicating better overall efficacy of labetalol. Our findings also build up study on findings by Khan et al where intravenous labetalol lowered mean arterial pressure (MAP) more effectively than hydralazine in pregnant women with this complicated hypertensive disorders, we used an escalating dose regimen for labetalol and more frequent BP monitoring, allowing for a more comprehensive evaluation of treatment response. We also assessed additional secondary outcomes such as failure rates and adverse effects, providing a broader understanding of medication safety.^16^

The inclusion of subgroup-Analyses based on age, parity, and BMI adds valuable insights, allowing for more personalized treatment recommendations. This enhances the generalizability of the findings to diverse patient populations. A study by Okai DE et al. (2020) also demonstrated the significance of demographic factors in influencing antihypertensive drug efficacy.^20^

Our study revealed that hydralazine achieved quick control of blood pressure than labetalol, aligning with research showcased at the 2019 Annual Meeting of the American College of Obstetricians and Gynecologists that suggest its rapid action in hypertensive emergencies.^21^ However, conflicting evidence exists, with other studies reporting faster BP control with labetalol.^22^ These variations may be due to differences in study populations, treatment protocols, and drug mechanisms. Hydralazine may be preferred for rapid BP reduction, while labetalol offers sustained control with fewer adverse effects. Khan SM et al. (2023) shared more incidence of hypotension in mothers and fetal heart rate abnormalities with hydralazine, suggesting labetalol as a safer option. Individualized treatment selection is crucial, and more comprehensive investigation is indispensable to ascertain the corroborate antihypertensive choice in severe gestational hypertension.^13^

A recent randomized controlled analysis by Otutoaja et al. (2023) compared the efficacy of labetalol and hydralazine in managing hypertension in pregnant ladies. The study found that labetalol was effective with patients requiring less doses compared to those receiving hydralazine. Additionally, side effects was proportionate between the two groups.^23^ Kausar et al. (2023) also found that labetalol achieved better blood pressure control with fewer doses compared to hydralazine. A systematic review showed that both drugs are effective in control of blood pressure and labetalol is better in reducing risk of maternal hypotension.^15^ However meta-analysis in 2018 documented labetalol as an effective alternative drug for treatment of severe hypertension.^24^ Evidence reported adverse effects with labetalol but comparable to hydralazine. Literature has not proven any medicine superior to other instead tailored recommendations are appreciated to improve maternal and fetal outcome.^25^

A major strength of this study is the well-defined cohort of women with severe gestational hypertension, allowing a focused comparison of two commonly used antihypertensive agents in a real-world tertiary care setting. The study also provides valuable subgroup analyses based on age, parity, and BMI, which is clinically relevant predictors of treatment response. Additionally, prospective data collection, predefined inclusion and exclusion criteria, and consistent follow-up strengthen the methodological rigor of the study. These strengths collectively make the findings more robust and clinically applicable for improving emergency management protocols in hypertensive disorders of pregnancy.

However, future research should focus on large, multicenter randomized controlled trials to validate the comparative efficacy of labetalol and hydralazine in diverse obstetric populations. Studies evaluating long-term maternal outcomes, neonatal morbidity, and the impact of repeated antihypertensive exposure during pregnancy are also needed. Comparative effectiveness studies across various levels of healthcare may further help standardize protocols in resource-limited settings.

### Limitations:

Although the advantages of this research, such as a clearly characterized patient Group-And rigorous statistical evaluation, some constraints are recognized. The non-randomized design introduces potential selection bias, and limited assessment of ongoing effects. Additionally, exclusion of high-risk pregnancies and reliance on subjective assessment of adverse effects may impact result interpretation.

## CONCLUSION

This study demonstrates that both IV labetalol and IV hydralazine are effective in managing severe gestational hypertension. Labetalol provides better overall efficacy and sustained blood pressure control, while hydralazine achieves faster BP reduction with fewer doses but a higher incidence of adverse effects. Individualized treatment selection based on patient characteristics and clinical urgency is crucial to optimize maternal and fetal outcomes. Further large-scale studies are needed to refine management strategies.
